# Comparison of CT and MR imaging in ischemic stroke

**DOI:** 10.1007/s13244-012-0185-9

**Published:** 2012-09-29

**Authors:** Josef Vymazal, Aaron M. Rulseh, Jiří Keller, Ladislava Janouskova

**Affiliations:** 1 Department of Radiology, Na Homolce Hospital, Prague, Czech Republic; 2 Department of Neurology, 1st Medical Faculty, Charles University in Prague, Prague, Czech Republic; 3 1st Medical Faculty, Charles University in Prague, Prague, Czech Republic; 4 3rd Medical Faculty Prague, Charles University in Prague, Prague, Czech Republic; 5 Na Homolce Hospital, Roentgenova 2, 15030 Prague 5, Czech Republic

**Keywords:** Cerebrovascular stroke, Magnetic resonance imaging, Computed tomography, spiral, Perfusion imaging

## Abstract

**Background:**

Cerebrovascular disease represents a major source of global mortality and morbidity. Imaging examinations play a critical role in the management of stroke patients, from establishing the initial diagnosis to determining and guiding further treatment.

**Methods:**

In this article, current CT and MRI methods employed in the management of stroke patients are reviewed, with an emphasis on ischemic stroke.

**Results:**

The advantages and disadvantages of these techniques are discussed, a number of cases emphasizing key points are presented, and a comparison between modern CT and MRI techniques is outlined.

**Conclusion:**

The major drawback of CT is the high radiation dose, while in MRI it is the more complicated and time-consuming aspect of the examination.

***Main Messages*:**

• *Cerebrovascular disease represents a major source of global mortality and morbidity*

• *Imaging examinations play a critical role in the management of stroke patients*

• *The penumbra may be seen with both CT and MRI; however, this concept may be overly simplistic*

• *The major drawback of CT is the high radiation dose, while MRI is a more complicated examination*

## Introduction

Cerebrovascular disease represents a major source of global mortality, with over 6 million deaths documented annually, and is the second leading cause of death in all income groups worldwide, exceeded only by ischemic heart disease [1]. In addition to being a leading source of mortality, cerebrovascular disease is also a significant cause of morbidity. As many as 50 % of stroke survivors do not regain functional independence, and 20 % require institutional care 3 months after stroke onset [2].

Stroke, or cerebrovascular accident, is characterised by the onset of neurological symptomatology that most often includes hemiparesis, aphasia or hemianopia (paraparesis is not typical for stroke and suggests an ischemic lesion in the spinal cord or other non-ischemic etiology). The onset may be sudden or discovered after arousal, and may be accompanied by headache, vomiting, vertigo or loss of consciousness. Stroke can be generally classified as ischemic or hemorrhagic. Treatment strategies for these two subtypes of stroke are markedly different, and the early diagnosis of stroke as well as determination of the subtype is an important early step in stroke management.

Hemorrhagic stroke, or intracerebral hemorrhage, represents 10–15 % of stroke cases. Although the incidence of this type of stroke is low, it is associated with significant morbidity and mortality. Up to 38 % of patients that experience hemorrhagic stroke will die within 30 days [3], and approximately half of survivors will remain dependent on others for activities of daily living [4]. Ischemic stroke is more common, representing approximately 85 % of all stroke cases, and has a much lower 30-day mortality rate at approximately 12 % [2]. Morbidity in ischemic stroke may also be severe and is highly dependent upon timely diagnosis and initiation of treatment. An ischemic lesion may also undergo hemorrhagic transformation. This is especially typical for infarction caused by venous occlusion. When hemorrhagic infarction is detected, occlusion of the venous (sinus) system should be considered. Additionally, transient ischemic attack (TIA) may occur, which involves focal neurological deficit that resolves within 24 h. Although self-limited, TIA may complicate the diagnosis of ischemic stroke. A TIA is also a strong short-term risk factor for stroke, as up to 20 % of patients that have a TIA will suffer a stroke within 90 days [2].

The expression “time is brain” relates to the time-dependent outcome in stroke management, and reflects the fact that the final infarct volume in ischemic stroke is dependent not only on regional cerebral blood flow (rCBF, ml/100 mg/min), but also on the length of time that rCBF has been reduced. There have been efforts to establish certain rCBF thresholds for functional and irreversible impairment in brain ischemia. Generally, rCBF < 50 is considered hypoperfusion, with oligemia occupying the 20–50 range, and ischemia < 20. The border between oligemia and ischemia is that point at which functional impairment manifests, called the functional threshold, and has been experimentally determined in a number of controlled animal studies [5, 6]. The point at which functional impairment becomes irreversible impairment, or infarction, is more difficult to define clearly and is related to the depletion of intracellular ATP and the subsequent loss of membrane integrity. The threshold for infarction may also be modulated by additional factors, such as changes in glucose metabolism leading to an increase of lactate, the influx of Ca^2+^ ions into the cell and the release of excitatory amino acids [7]. Recently, the role of glutamate in the development of the final ischemic volume has been emphasised. The release of large amounts of glutamate may lead to cortical spreading depression (CSD) and peri-infarct depolarizations (PID) that further tax the energy capabilities of ischemic cells [8, 9]. It has been experimentally proven that during the first 3 h after ischemia, each depolarization increases the infarct volume by roughly 20 % [10]. Conversely, a history of TIA has been shown in some studies to impart a certain degree of neuroprotection, likely gained as a lasting adaptive cellular response to ischemia [11, 12].

Regardless of modulating factors, the development of irreversible ischemia remains highly time dependent. An rCBF of 17–18 may be reversible at durations approaching infinity, while an rCBF < 9 likely represents the lower border of reversibility within an interventional time frame that is realistically achievable in the clinical setting [13]. The zone positioned between the functional threshold and the infarction threshold is called the penumbra, and is considered to be the volume of ischemic tissue that can potentially be salvaged if reperfused. As time elapses, the infarction threshold increases and the penumbra decreases in volume. In more practical terms, while an rCBF of < 9 will in many cases lead to infarction within 20–30 min, at 60 min the threshold may be an rCBF < 12, and at 45 min < 14. Therefore, the successful management of ischemic stroke is dependent upon establishing the diagnosis and initiating treatment as quickly as possible.

Thrombolytic agents are the treatment of choice in ischemic stroke, within certain limits. Although temporal guidelines for the administration of these agents vary by country and region, 3 h from the known onset of clinical manifestations to the time of treatment is universally considered a safe and effective interval. Preconditions for thrombolytic treatment include, but are not limited to, the exclusion of hemorrhage and the demonstration of salvageable tissue. Therefore, characterisation of the penumbra plays a vital role in the workup of ischemic stroke. Additionally, characterization of the penumbra may allow a more individualised treatment approach. The 3-h interval between manifestation and treatment was arrived at following large randomised trials and may in some cases be too restrictive [14, 15]. It has been shown that patients with certain perfusion characteristics may benefit from thrombolytic treatment beyond the 3-h window [16–19]. Ideally, studies such as these would enable clinicians to make treatment decisions based upon objective findings unique to the patient rather than arbitrary time constraints. This would be of obvious benefit to the large group of patients that present with an unknown time of onset. However, there are many pitfalls and confounders that complicate relying solely on imaging findings, as will be discussed below.

## Imaging

**Fig. 1:**
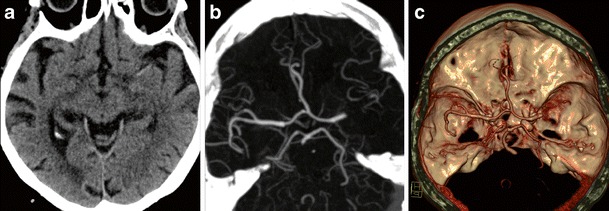
Acute obliteration of the left MCA in an 82-year-old female. **a** Hyperdense artery sign suggesting an acute thrombus in the left MCA. **b** CT angiography shows obliteration of the left MCA. **c** VRT reconstruction. MCA, middle cerebral artery; VRT, volume-rendering technique

**Fig. 2:**
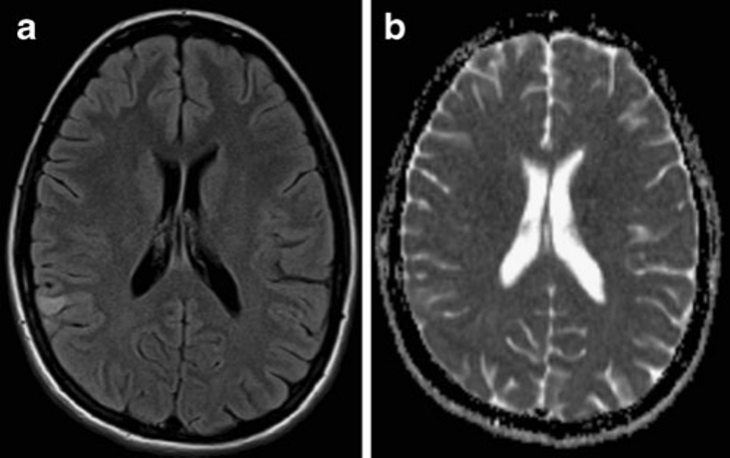
A 20-year-old female with sudden onset of neurological symptomatology. **a** FLAIR image shows an area of increased signal intensity in the right angular region. **b** ADC map does not show restricted diffusion that would be characteristic for an acute ischemic stroke. Encephalitis was proven by CSF examination. FLAIR, fluid attenuated inversion recovery; ADC, apparent diffusion coefficient; CSF, cerebrospinal fluid

**Fig. 3:**
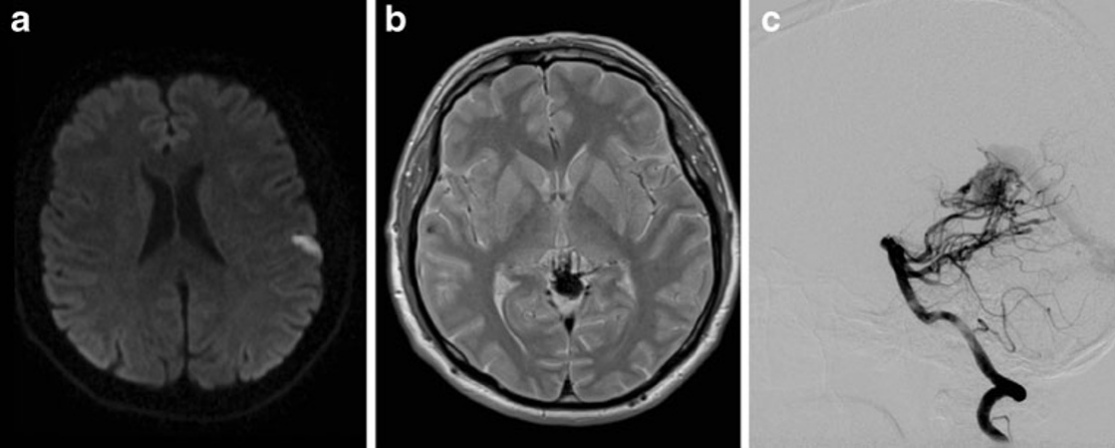
A 55-year-old male with sudden onset of transitory aphasia. **a** DWI (b-factor 1000) shows an area of restricted diffusion in the left parietal region. **b** PD-weighted SE image shows an AVM in the pineal region. **c** DSA proves a combined pial-dural AVM. Although the precise pathogenesis of acute neurological symptomatology in a patient with an AVM is unknown, the “steal phenomenon” may play a role in its development. DWI, diffusion-weighted imaging; PD, proton density; SE, spin echo; AVM, arteriovenous malformation; DSA, digital subtraction angiography

**Fig. 4:**
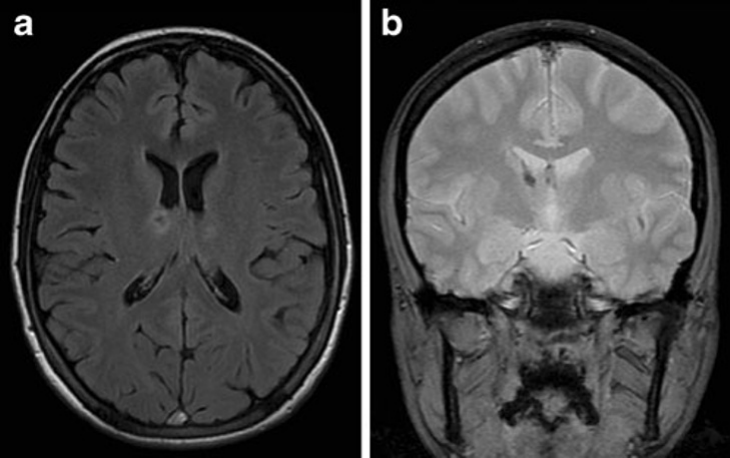
Acute hemorrhagic infarction in the right thalamus due to venous sinus occlusion in a 30-year-old woman with a history of hormonal contraception. **a** FLAIR image and **b** gradient-echo T2*-weighted image proving hemorrhagic transformation. FLAIR, fluid-attenuated inversion recovery

A number of clinical tests have been developed over the years to help determine the presence of stroke [e.g., 20, 21]. Although these tests may aid in the initial triage of acute neurological patients, they cannot match the sensitivity and specificity of an imaging examination, nor is there any clinical test available that can accurately differentiate ischemic and hemorrhagic stroke [22]. Therefore, the initial step in the management of a suspected stroke patient is an imaging examination. A non-contrast computed tomography (CT) examination, often employed at this stage, can quickly exclude the presence of hemorrhage. The absence of hemorrhage supports the diagnosis of an ischemic event, and some evidence of ischemia may be seen in the native CT as well. The hyperdense vessel sign and signs related to the loss of contrast between the gray and white matter (such as the insular ribbon sign and lentiform obscuration) are all examples of signs of acute ischemia on native CT (Fig. 1). The imaging examination also serves to exclude other pathologies that may resemble stroke clinically, known as the “stroke mimics.” Such pathologies include, but are not limited to, spinal stroke, hemorrhagic neoplasms, encephalitis, multiple sclerosis, postictal (Todd’s) paresis, some types of migraine, intoxications, hypertensive encephalopathy, hyper- or hypoglycemia, as well as psychiatric diseases (Fig. 2). Ischemic events, often of transitory duration, may also have their origin in arteriovenous malformations. A steal phenomenon may be responsible for the symptomatology in these cases although this concept is controversial (Fig. 3) [23]. Additionally, the distinction between ischemic stroke following arterial occlusion and ischemia following venous sinus occlusion with secondary hemorrhage is important. The treatment of venous occlusive disease is different from primary stroke, and the prognosis of the patient fully depends upon early diagnosis (Fig. 4). Magnetic resonance imaging (MRI) is usually more sensitive and specific in distinguishing both the stroke mimics and secondary ischemic lesions. Finally, the application of a contrast agent may increase the specificity of imaging. Especially contrast-enhanced MRI can reveal a typical cortical pattern of ischemic enhancement and/or help to detect other pathologies that belong to the stroke mimics.

## CT and MR angiography

Imaging the vessels, the origin of all stroke pathology, is an essential part of the stroke protocol and follows the initial native CT. Imaging of the vertebral and carotid arteries, preferably from the aortic arch to the circle of Willis, its major branches and the initial branches beyond, should be covered. Angiography improves diagnostic precision, providing insight into the source of dysfunction, and may lead the management towards intervention [24, 25]. Both CT and MR angiography may be used, each maintaining some advantages and disadvantages.

When the state-of-the-art CT scanners are employed, a single bolus of contrast agent is sufficient to achieve both angiography and perfusion images. Thus, CT angiography requires the application of a contrast agent, but its resolution is always better than that of MR angiography. However, in detecting the occlusion of a larger artery such as the middle carotid artery (MCA), both CT and MR angiography are usually straightforward (Fig. 1). MCA occlusion is the most common target of interventional stroke treatment [26]. MR brain angiography in the stroke protocol is non-contrast in most cases and is based on the inflow of fresh spins into the imaging slice, called the time-of-flight (TOF) technique. One limitation of MR angiography is that the aortic arch is not covered. Another non-contrast technique, phase-contrast angiography, is rarely used in stroke management, but is a valuable tool in detecting venous sinus thrombosis, which often leads to stroke. CT angiography source data may also be used for an estimation of the final infarct volume, similar to DWI in MRI. Furthermore, using this technique it is possible to distinguish patients at risk of infarct growth according to the status of the collateral circulation [27].

## Perfusion imaging

**Fig. 5:**
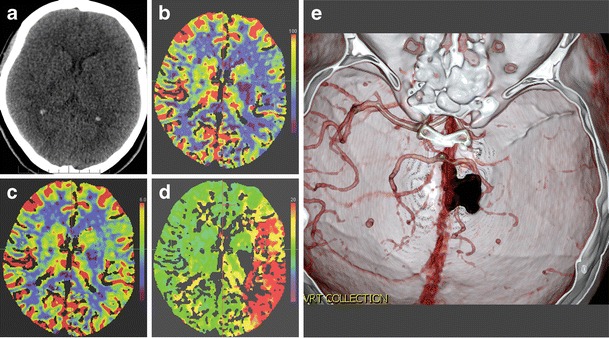
Acute ischemic stroke due to obliteration of the left ICA and MCA. **a** Non-contrast CT is practically normal, with no hypodense areas and no mass effect present. **b** CBF map shows hypoperfusion (colder colours) in the region of the left MCA. **c** CBV map with only subtle differences between left and right hemispheres. **d** TTP map displays a significant delay in the region of the left MCA due to collateral flow. **e** CT angiography displays obliteration of the left MCA. ACI, internal carotid artery; MCA, middle cerebral artery; CBF, cerebral blood flow; CBV, cerebral blood volume; TTP, time-to-peak

The goal of perfusion imaging is not only the diagnosis of ischemia, but also the detection of the penumbra. In order to quantify and more precisely detect brain perfusion, several standard flow parameters are calculated (Fig. 5). Mean transit time (MTT, s) represents the mean time required for a volume to clear the capillaries, while the time-to-peak (TTP, s) reflects the time required for a volume to reach peak concentration. Both MTT and TTP are very sensitive to local perfusion disturbances, but less specific to ischemia or infarction [28]. Cerebral blood volume (CBV, ml/100 g brain) represents the volume of blood in a volume of tissue, and reflects autoregulation. As perfusion pressure decreases, autoregulatory mechanisms are activated, locally resulting in vasodilatation and recruitment of supporting capillary networks to increase perfusion of the ischemic region. The results of these changes are increased CBV, MTT and TTP. Within the ischemic core there is a failure of autoregulation, and CBV is ominously decreased in this region. Cerebral blood flow (CBF, ml/100 g brain/min) represents the delivery of blood to tissue per unit time and is calculated by dividing the CBV by MTT. CBF is decreased in all hypoperfused regions, including both the penumbra and ischemic core. On perfusion CT, CBV has been shown to correlate with infarct volume [29], and the subtraction of CBF and CBV is the usual way to detect the penumbra. With MRI, diffusion-weighted imaging (DWI) is considered to correlate with infarct volume [30], and subtraction between CBF and DWI is used to demonstrate the penumbra. A comparison between MR and CT perfusion is presented in Table 1.

**Table 1:** Comparison between MR and CT perfusion

CT	MRI
Detection of hemorrhage	Detection of hemorrhage with SWI
Angiography, better resolution	Angiography, non-contrast
Faster, more available, less restrictive	Slower, limited availability, restrictive environ
Radiation	No radiation
Limited in posterior fossa	Better detection in posterior fossa
Limited detection of small lesions	Better detection of small lesions (DWI)
Less specific in detection of “stroke mimics”	Better detection of “stroke mimics,” especially on contrast-enhanced scans

*SWI* susceptibility-weighted imaging, *DWI* diffusion-weighted imaging

## Perfusion computed tomography (PCT)

The introduction of perfusion computed tomography (PCT) of the brain significantly improved the sensitivity and specificity in detecting fresh ischemia [28]. Perfusion deficit can be detected immediately after stroke, i.e., in the time when a standard CT is still negative (Fig. 5). A major disadvantage of this technique is the limited coverage of the brain with older scanners. In such cases the coverage is limited to several slices, and the neurologist or neuroradiologist has to decide which part of the brain should be covered. It may happen that misleading neurological symptomatology leads the physician to set the perfusion slab in the wrong position. An example may be setting the covered area around the basal ganglia with the ischemia located in the upper subcortical/cortical areas, or ischemia in the posterior fossa without cerebellar symptomatology or alternating hemiparesis, thus mimicking supratentorial involvement. Therefore, 75-mm brain coverage is the minimal PCT coverage recommended in selecting patients with acute stroke for reperfusion therapy [31]. However, the emergence of state-of-the-art CT scanners enables coverage of almost the whole brain and has resolved this limitation.

Another drawback of CT is the high radiation dose. CT is considered to be a major source of radiation in developed countries [32]. Although ultrafast CT scanners together with iterative reconstructions can significantly reduce the radiation dose in some applications, such as abdominal or cardiac CT, in perfusion CT of the brain the dose still remains relatively high (mean effective dose is approximately 5 mSv) [33]. Additionally, the long reconstruction times of iterative techniques, in the range of minutes, are inconvenient for stroke patient management. There are however techniques that are able to decrease the radiation dose to values comparable to unenhanced brain CT (approximately 2 mSv). A decrease from 140–120 kV to 100–80 kV not only significantly lowers the radiation dose, but also increases the prominence of the contrast agent because of the greater importance of the photoelectric effect for 80-kV photons [34]. Another method for dose reduction relies on decreasing the image frequency of acquisition from the PCT first-pass data [35].

PCT enjoys a number of advantages as it is fast, widely available, and cost effective. Furthermore, there are few restrictions in the CT environment. However, CT is less sensitive and less specific in the detection of ischemia than MRI. Other lesions, such as acute demyelinating plaques, inflammatory lesions and some brain tumors, may mimic brain ischemia on CT [36]. A number of pitfalls need to be considered in performing PCT as well. The operator-dependent selection for the arterial input function may have a substantial influence on the results, especially in subjects with chronic vascular conditions such as giant aneurysm or carotid artery occlusion, as well as in patients with asymmetric microvascular changes [37]. Postictal changes are another concern, as lateralised postictal hyperperfusion may lead to the erroneous diagnosis of contralateral ischemia on visual inspection of colour maps [38]. Furthermore, postprocessing and modeling methods may affect the results and may complicate comparison across studies [39].

## MR perfusion

Although there has been a great deal of interest in using MRI in the acute workup of stroke, it remains largely academic. MRI is known to generally be more sensitive than CT in the detection of ischemia, and current experimental MRI studies with Na^23^ show even better sensitivity for acute stroke imaging [40]. However, the detection of hemorrhage, especially smaller hemorrhage, is not so straightforward with MRI. Diamagnetic oxyhemoglobin present in fresh hemorrhage only changes the proton density, the least sensitive MRI parameter, and not the relaxation times that are primarily responsible for signal intensity on MR images. This disadvantage has since been balanced with the introduction of susceptibility-weighted imaging (SWI), a technique that is able to detect even small amounts of paramagnetic deoxyhemoglobin, which is always present in fresh hemorrhage [41]. However, other disadvantages of MRI in the management of stroke patients remain, such as its relatively longer duration and more restrictive environment. Patients that are disorientated or unconscious cannot provide a reliable personal history regarding the presence of any implanted devices or prostheses.

**Fig. 6:**
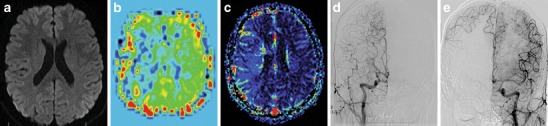
A 34-year-old male with a history of two transient ischemic attacks and normal neurological examination. Standard MRI was normal. **a** DWI (*b* = 1,000). **b** PASL sequence shows hypoperfusion in the distribution of the right MCA. **c** Contrast perfusion is concordant with PASL. DSA shows obliteration of the right MCA with very good collateral flow from the **d** ipsilateral and also **e** contralateral vascular territories. DWI, diffusion-weighted imaging; PASL, pulsed arterial spin labeling; MCA, middle cerebral artery; DSA, digital subtraction angiography

**Fig. 7:**
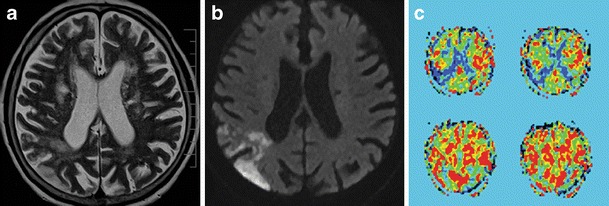
An 86-year-old male with a history of previous ischemic events developed sudden left-sided hemianopia. **a** T2-weighted TSE image with a number of ischemic lesions. **b** DW image (*b* = 1,000) clearly shows a region of acute ischemia as an area of restricted diffusion. **c** PASL image with a perfusion deficit in the right occipital region. TSE, turbo spin echo; DW, diffusion weighted; PASL, pulsed arterial spin labeling

**Fig. 8:**
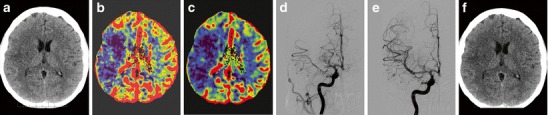
A 41-year-old obese, normotensive female treated with hormonal contraception developed left-sided hemiplegia at 6 a.m. **a** Acute CT at 7:30 a.m. shows a mild hyperdense lesion in the distribution of the right MCA and a large perfusion deficit with no penumbra on **b** CBF and **c** CBV maps. At 8:20 a.m. intravenous Actilyse was applied, and at 10:35 a.m., despite the absence of penumbra, **d** mechanical thrombolysis of the obliterated right MCA was performed **e** with successful thrombus removal. Left-sided hemiplegia resolved within a week. Six weeks after the stroke the patient was fully independent, going to work, driving a car and feeling no limitation of her left extremities except for “slight clumsiness” of her left hand. **f** CT scan with minimal residual ischemic changes. MCA, middle cerebral artery; CBF, cerebral blood flow; CBV, cerebral blood volume

The major advantages of MRI are whole brain coverage with a standard scanner, absence of radiation and relatively safe contrast applied in lower doses, between approximately 10–20 ml (Fig. 6). In addition, a recent MRI technique called arterial spin labeling (ASL) enables basic perfusion imaging without the administration of a contrast agent. This technique, which enables the quantitative measurement of CBF, is based on magnetic labeling of blood prior to flowing into a volume of interest, typically in the neck (i.e., in the common carotid arteries). An inversion pulse is used to tag inflowing spins proximal to the imaging slab, and, following a certain delay to allow these spins to enter the imaging slab and exchange with protons in the tissue, control and label images are obtained [42]. A subtraction technique is then used for the final image calculation (Figs. 7, 8). Signal change caused by spin labeling is directly proportional to blood flow (CBF increase of ∼1 ml/100 g tissue/min increases signal by ∼1 %); therefore, CBF data can be easily calculated [43]. Labeling can be either continuous (CASL) or pulsed (PASL). In CASL, blood is labeled even during the readout phase. This approach requires a special labeling coil and has a higher signal-to-noise ratio (SNR) with a penalty of higher specific absorption rate (SAR) [44]. Therefore, PASL is more commonly used at 1.5 T and 3 T. With this approach, a saturation pulse is followed by a short delay, and then an echo-planar image of an area of interest is acquired. PASL benefits from higher SNR and T1 prolongation at 3 T. Several acquisition schemes for PASL exist (with QUIPPS II being commonly used) and are available as products from major vendors. The drawbacks of ASL are longer acquisition times and measurement of CBF only, as with ASL it is currently not possible to calculate MTT, TTP or CBV. The main advantage is the ability to perform imaging without the use of contrast, which can be crucial in subjects with known allergy, high risk of nephrogenic systemic fibrosis or in longitudinal studies. Although ASL and SWI improve the utility of MRI in stroke management, in many centres these techniques are not yet part of routine clinical practice.

## Penumbra detection

Different parameters are used in routine clinical practice for the detection of penumbra with CT and MRI. The subtraction of CBF and CBV is the usual way to detect penumbra using CT. On MRI, a subtraction between perfusion parameters (MTT, TTP or CBF) and DWI, rather than CBV, is used (Fig. 7). This is due to the fact that CBV (especially on MRI) is not sensitive enough to detect small lesions below approximately 10 ml. Such lesions are easily detected with DWI [45]. DWI, on the other hand, may underestimate the final stroke volume and grows into CBV over the time [46].

The penumbra concept is far from straightforward, however, as a number of different factors determine whether the tissue will become necrotic, such as the time-dependent nature of morphological changes discussed previously. The situation is even more complicated because exact quantification of perfusion values (CBF, CBV) is rarely used in routine clinical practice. Thus, relying only on CBF/CBV mismatch might disqualify some patients that would benefit from intervention (Fig. 8). Several studies have shown that even in critically hypoperfused tissue, only a part of the predicted final infarct volume became necrotic after thrombolytic therapy [47, 48]. For these reasons, no quantitative parameters related to the penumbra should currently be taken for granted, and neuroimaging of the penumbra based on the presence of mismatch on CT or MRI may bring ambiguous results in relation to treatment outcome [7].

## Conclusions

In comparing the use of CT versus MRI in the workup of ischemic stroke, a number of factors come under consideration. When ultrafast CT scanners covering almost the entire brain are used, the potential to detect ischemia and salvageable tissue is almost equal in both techniques. The major drawback of CT is the high radiation dose, while in MRI it is the more complicated and time consuming aspect of the examination. Thus, if any general practical recommendation can be made, acute stroke patients should be evaluated by native CT followed by PCT, with MRI reserved for more chronic cases of brain ischemia or control examinations in stroke patients. However, when state-of-the-art CT and high-quality MR scanners are utilised, both techniques are practically equivalent in the hands of experienced personnel, and local clinical configurations may often dictate which technique should be used.
